# Determinants of perceived health and unmet healthcare needs in universal healthcare systems with high gender equality

**DOI:** 10.1186/s12889-021-11531-z

**Published:** 2021-07-31

**Authors:** Christina P. Tadiri, Teresa Gisinger, Alexandra Kautzky-Willer, Karolina Kublickiene, Maria Trinidad Herrero, Colleen M. Norris, Valeria Raparelli, Louise Pilote, on behalf of the GOING-FWD Consortium

**Affiliations:** 1 Research Institute of McGill University Health Centre, Division of Clinical Epidemiology McGill University, Montreal, Canada; 2 Department of Medicine III, Division of Endocrinology and Metabolism, Medical University of Vienna, Vienna, Austria; 3 Department of Renal Medicine, Institution for Clinical Science, Intervention & Technology, Karolinska Institute, Stockholm, Sweden; 4 Clinical and Experimental Neuroscience (NiCE), Institute for Aging Research, Institute for Bio-Health Research of Murcia (IMIB), School of Medicine, University of Murcia, Murcia, Spain; 5 Faculty of Nursing, University of Alberta, Edmonton, Canada; 6 Heart Health & Stroke, Strategic Clinical Network-Alberta Health Services, Edmonton, Alberta Canada; 7 Department of Translational Medicine, University of Ferrara, Ferrara, Italy; 8 University Center for Studies on Gender Medicine, University of Ferrara, Ferrara, Italy

**Keywords:** Social determinants of health, Patient-reported outcomes, Public health, Country/cultural determinants of health

## Abstract

**Background:**

Patient attitudes about health and healthcare have emerged as important outcomes to assess in clinical studies. Gender is increasingly recognized as an intersectional social construct that may influence health. Our objective was to determine potential sex differences in self-reported overall health and access to healthcare and whether those differences are influenced by individual social factors in two relatively similar countries.

**Methods:**

Two public health surveys from countries with high gender equality (measured by UN GII) and universal healthcare systems, Canada (CCHS2014, *n* = 57,041) and Austria (AT-HIS2014, *n* = 15,212), were analysed. Perceived health was assessed on a scale of 1 (very bad) to 4 (very good) and perceived unmet healthcare needs was reported as a dichotomous variable (yes/no). Interactions between sex and social determinants (i.e. employment, education level, immigration and marital status) on outcomes were analysed.

**Results:**

Individuals in both countries reported high perceived health (Scoring > 2, 85.0% in Canada, 79.9% in Austria) and a low percentage reported unmet healthcare needs (4.6% in Canada, 10.7% in Austria). In both countries, sex and several social factors were associated with high perceived health, and a sex-by-marital status interaction was observed, with a greater negative impact of divorce for men. Female sex was positively associated with unmet care needs in both countries, and sex-by-social factors interactions were only detected in Canada.

**Conclusions:**

The intersection of sex and social factors in influencing patient-relevant outcomes varies even among countries with similar healthcare and high gender equality.

**Supplementary Information:**

The online version contains supplementary material available at 10.1186/s12889-021-11531-z.

## Background

A primary goal of public health is to identify vulnerable groups or pathways through which individuals experience poor health. Social determinants of health are known to play a large role in health outcomes, mediated through lifestyle factors which may influence an individual’s likelihood of developing chronic conditions. For example, social determinants may impact an individual’s ability to access nutritious food and healthcare resources, time and space for physical activity [1, 2] as well as an individual’s mental health through chronic financial or psychosocial stress [3], which may influence an individual’s overall perception of their health. Self-reported health has been shown to often correspond to an individual’s life expectancy or presence of comorbidities [4, 5] and also positively affects self-reported health [6]. Therefore, understanding people’s lived experiences and their perception of health and access to healthcare, and associations with social and lifestyle factors as well as biological factors such as sex is important to improving health equity.

Many traditional psychosocial determinants of health are gendered, and therefore may impact health outcomes differently for men, women and gender-diverse individuals through various pathways. Gender is a complex social construct defined by four domains: gender identity, gender roles, gender relations and institutionalized gender [7]. Gender intersects with race, ethnicity, indigenous status, sexuality, geography, age, disability/ability, migration status, socioeconomic status and religion [8–10] with a suggested interactive effect on the individual perception of health and healthcare [11, 12]. Although many studies have independently assessed social determinants of health, few have explored their interaction with sex to assess the gendered impact of these factors. Furthermore, conceptions of gender often vary over time and with culture, as may their influence on health and few studies have examined the intersection of sex, and social determinants of health across countries to incorporate this cultural impact.

The objective of this study was to determine if there were sex differences in self-reported overall health and access to healthcare and whether those differences are influenced by individual social factors such as income, education level, working status, marital status and migration history in two countries with publicly funded healthcare and relative gender equality (Canada and Austria). We hypothesized that, due to the intersection of gender with many other social variables, the influence of these social factors on health would vary by sex. The secondary objective of this study was to determine whether the influence of these factors differed between countries. We hypothesized that due to differences in cultural perceptions of gender and policies, these relationships would differ.

## Methods

### Data source

As part of the GOING-FWD Consortium (https://www.mcgill.ca/going-fwd4gender/), this study leveraged data from two large, national public health surveys: the Canadian Community Health Survey (CCHS) and the Austrian Health Information Survey (AT-HIS).

A full description of the methodology of both surveys can be found on their websites [13, 14]. Briefly, AT-HIS has been administered twice (in 2007 and 2014) as part of the larger European Health Information Survey and consists of individuals over the age of 15, who are living in Austria and were randomly chosen and asked about their health, their lifestyle and their utilization of the healthcare system. AT-HIS data were obtained from Statistik Austria after providing a brief description of the project. Statistics Canada has run the CCHS biennially for several years. The CCHS is a large randomized cross-sectional survey of people over the age of 12 living in Canada asking basic demographic information, health, lifestyle, and utilization of healthcare systems questions. Public Use Microdata Files (PUMF) derived from the master files to ensure respondent security are available for researchers that remove or transform variables that could lead to individual identification. Both surveys were comprehensive assessing demographic, lifestyle and social variables as well as overall health and chronic conditions and healthcare utilization, recruiting a broad range of residents of each country to serve as a representative sample of the country.

For maximum compatibility, we selected data from the 2014 round of each cohort, removed participants under the age of 20 (CCHS *n* = 57,041; AT-HIS *n* = 15,212) and data dictionaries were scanned for common questions that could be used in analysis. Baseline, social and outcome variables were identified, and the coding of variables were assessed to determine harmonization potential. Variables from each dataset were then harmonized to create datasets in which all variables were categorized in the same way.

### Exposure and outcomes

Baseline common variables included biological sex (male or female) and age (10-year brackets). Common social determinants included level of education (did not finish secondary school, completed secondary school, some post-secondary, completed post-secondary), level of income (low, middle and high as classified by respective tertials within each cohort), immigration status (born with Canadian citizenship or not for CCHS, born with Austrian citizenship for AT-HIS), working status (currently working or currently not working), marital status (single/never married, divorced/widowed, married/living with partner) and household size (continuous numeric variable).

Common reported outcomes of interest identified were perceived health overall on a scale of 1 (very bad/poor) – 4 (very good), and feelings of unmet healthcare needs (yes/no).

### Statistical analysis

Due to restrictions on sharing data between countries, we were unable to physically merge the harmonized datasets into one and instead conducted independent analyses of the harmonized datasets within each country and compared results. Missing data (0.2% for perceived health, 0.3% for perceived unmet care in CCHS, 0% for perceived health, 28.5% for perceived unmet care in AT-HIS) were removed from analyses.

For each country, logistic regression models were constructed for perceived health (bad = 1 or 2 vs. good = 3 or 4) and perceived unmet healthcare needs. First, univariate analysis was conducted to determine the associations between sex and all social determinants on outcomes.

For each outcome two different multivariable models were employed. The first full-adjusted model included the main independent effect of sex and social factors, in addition to other potential confounders (age), on each outcome. The second model included all significant variables, and a series of two-way sex-by-social variable interactions were tested to estimate whether the effect of each social factor varied based on sex. All data harmonization and analysis were conducted in R version 4.0.0 and in all cases α = 0.05.

## Results

A total of 25,044 men and 31,997 women were included in CCHS and a total of 6713 men and 8499 women were included in AT-HIS.

The overall characteristics of surveyed participants are reported in Table 1. In both CCHS and AT-HIS, most participants were married or living with partner and the median household size was 2. In both countries, most participants were working, and had completed at least secondary education, with a higher proportion having completed post-secondary education in Canada than in Austria. The distribution of income differed between countries, with more Canadians being classified as middle income (49.5% versus 21% in Austria) and more Austrians being classified as high income (44% versus 24.1% in Canada). In both countries, most respondents were born with native citizenship, however the proportion of immigrants was higher in Canada (15.1%) than in Austria (5%).

**Table 1:** Overall cohort characteristics for Canada (CCHS) and Austria (AT-HIS)

	CCHS (%) *n* = 57,041	AT-HIS (%) *n* = 15,212
**Women (%)**	56.1	55.9
**High Perceived Health (%)**	84.9	79.9
**Perceived Unmet Care (%)**	10.9	10.7
**Working (%)**	61	59.9
**Single (%)**	20	27.7
**Married (%)**	23.3	62.2
**Divorced/Widowed (%)**	56.7	10.1
**Large household (median-split) (%)**	26	46.3
**Immigrant (%)**	15.1	5.1
**Lowest income**	26.4	35.3
**Highest income (%)**	24.1	43.5
**Did not Complete Secondary School (%)**	18.1	13.9
**Completed Post-Secondary School (%)**	57.2	33.3

In both countries, the majority of respondents rated their overall health as either good or very good (85% in Canada and 79.9% in Austria) and the proportion of females reporting good health was higher than males in Canada, whereas it was lower than males in Austria. In both countries, the percentage of respondents reporting unmet healthcare needs was low (10.1% in Canada and 10.7% in Austria) and in both countries, the percentage of females reporting unmet healthcare needs was higher than for males.

The percentage of respondents reporting high or low perceived health, unmet care and social determinants varied by sex and country (Table 2). Additionally, male and female participants from both countries reporting high or low perceived health (Table 3) and perceived unmet care (Table 4) varied in their social determinants.

**Table 2:** Descriptive statistics of outcomes and social variables by sex and country

	Canadian Females ***N*** = 31,997	Canadian Males *N* = 25,044	Austrian Females *N* = 8499	Austrian Males *N* = 6713
**Perceived Health: 1 (very bad) (%)**	5	4.2	4	3,5
**Perceived Health: 2 (bad) (%)**	10	11.4	16.7	16
**Perceived Health: 3 (good) (%)**	30	32.1	42.1	44.7
**Perceived Health: 4 (very good) (%)**	55	52.3	37.2	35.8
**Perceived Unmet Care (Yes) (%)**	11.9	9.6	12	8.8
**Working (%)**	57	66	54.3	66.9
**Single (%)**	17.5	23.2	25.4	30.7
**Married (%)**	53	61.3	63.2	61
**Divorced/Widowed (%)**	29.5	15.4	11.4	8.3
**Household Size (%)**	2.1 (±0.006)	2.2 (±0.007)	2.66 (±0.013)	2.71 (±0.015)
**Immigrant (%)**	15	15.3	5.3	4.9
**Lowest income tercile**	32.9	18	38.3	31.6
**Highest income tercile (%)**	16.4	34	40	48
**Did Not Complete (%) Secondary School**	18	18.3	18.4	8.1
**Completed Post- Secondary School (%)**	56.8	57.8	31.3	35.9

*Data are presented as means ± standard error or percentages*

**Table 3:** Descriptive table of perceived health (low = 1,2; high = 3,4) by sex and social variables in Canada and Austria

	Canadian Females High Perceived Health (***N*** = 27,241)	Canadian Females Low Perceived Health (***N*** = 4701)	Canadian Males High Perceived Health (***N*** = 21,109)	Canadian Males Low Perceived Health (***N*** = 3894)	Austrian Females High Perceived Health (***N*** = 6739)	Austrian Females Low Perceived Health (***N*** = 1760)	Austrian Males High Perceived Health (***N*** = 5402)	Austrian Males Low Perceived Health (***N*** = 1311)
**Not Working (%)**	39.3	68.8	29.8	61	39.4	69.8	26.6	60.2
**Working (%)**	60.7	32.2	70.2	39	60.6	30.2	73.4	39.8
**Single (%)**	17.8	16.1	23.4	22	27.7	16.4	33.5	19.5
**Married (%)**	55.1	41.5	62.7	54.2	10.6	68.8	59.2	68.2
**Divorced/Widowed (%)**	27.1	42.4	13.9	23.8	61.7	14.8	7.3	30.2
**Large household (median-split) (%)**	27.2	14.9	29.5	16.3	49.4	31.8	50.3	33.1
**Small Household (%)**	72.8	85.1	70.5	83.7	50.6	68.2	49.7	66.9
**Immigrant (%)**	14.9	15.4	15.5	14.1	5.7	3.9	5.1	3.8
**Non-immigrant (%)**	85.1	84.6	84.5	85.9	94.3	96.1	94.9	96.2
**Lowest income (%)**	29.6	52.2	14.6	36.8	34.6	52.3	29.2	41.4
**Highest income (%)**	18.1	6.4	37.3	15.8	43.6	25.7	50.8	36.5
**Did not Complete Secondary School (%)**	15.1	34.6	15.5	33.4	13.8	36	6.7	14.3
**Completed Post-Secondary School (%)**	46.7	39.2	60.4	43.3	34.9	17.6	39	23

**Table 4:** Descriptive table of perceived unmet care by sex and social variables in Canada and Austria

	Canadian Females Unmet Care (***N*** = 3791)	Canadian Males Unmet Care (***N*** = 2394)	Austrian Females Unmet Care (***N*** = 767)	Austrian Males Unmet Care (***N*** = 398)
**Not Working (%)**	12.7	11.2	10.6	7.8
**Working (%)**	13.1	9.5	13.2	9.4
**Single (%)**	15.6	12.4	13.3	8.4
**Married (%)**	11.2	10.9	11.4	8.7
**Divorced/Widowed (%)**	10.6	1.7	12.4	10.9
**Large household (median-split) (%)**	13	9.5	12.6	9
**Small Household (%)**	11.5	9.6	11.5	8.7
**Immigrant (%)**	10.9	9.2	14.1	11.1
**Non-immigrant (%)**	12.1	9.7	11.9	8.7
**Lowest income (%)**	13.6	14.5	11.5	8.9
**Highest income (%)**	12.3	8.3	12.1	9.5
**Did not Complete Secondary School (%)**	9.2	8.8	16.2	7.5
**Completed Post-Secondary School (%)**	13.3	9.8	35.3	34.7

### Final models: perceived health overall

**Fig. 1:**
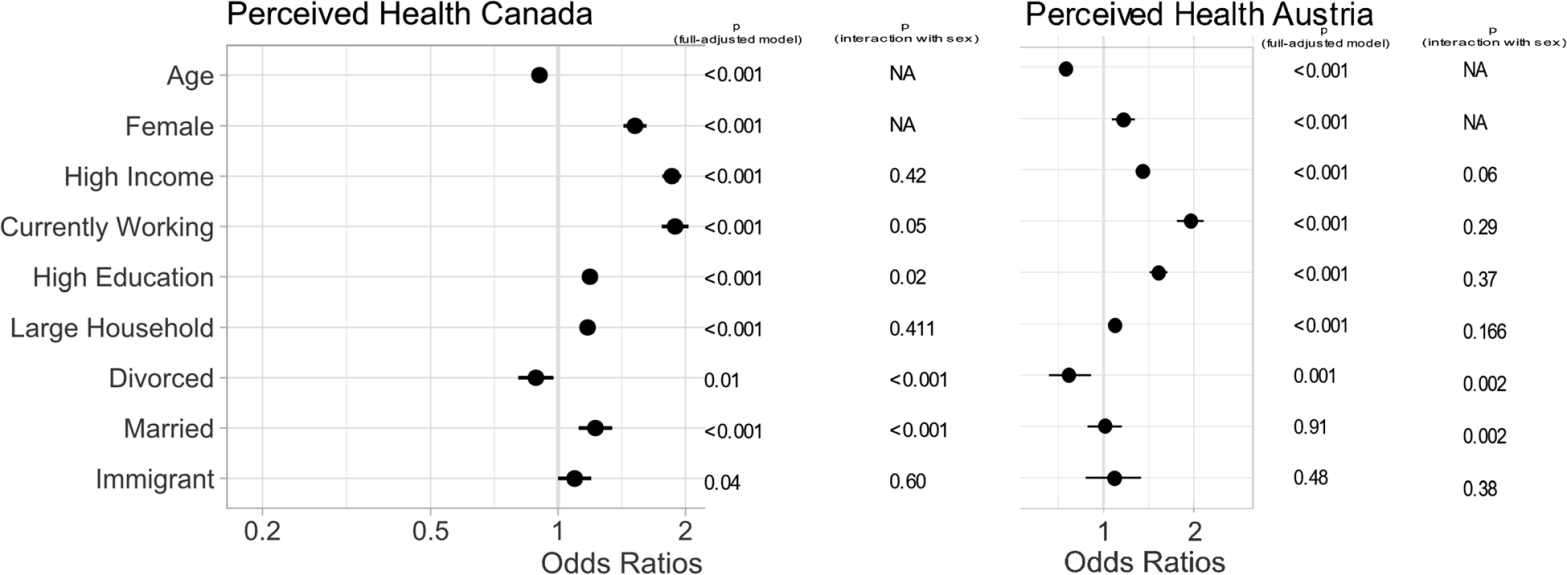
Forest plot of multivariate model for perceived health in Canada using sex and all social variables, adjusted for age and significance of sex x social variable interaction terms

In the fully-adjusted models, in both Austria and Canada, perceived health overall was positively associated with female sex, high education level, high income, working and large household size (all *p* < 0.001), and in only Canada, with immigration (*p* = 0.04) (Fig. 1). Additionally, a statistically significant sex-by-marital status interaction was detected in both countries (*p* < 0.01). For both sexes, being divorced or widowed significantly lowered perceived health overall, but the effect was more profound for males. Additionally, a sex-by-education level interaction was detected in Canada (*p* = 0.02), where the magnitude of high education level’s positive impact was greater in males.

### Final models: perceived unmet care

**Fig. 2:**
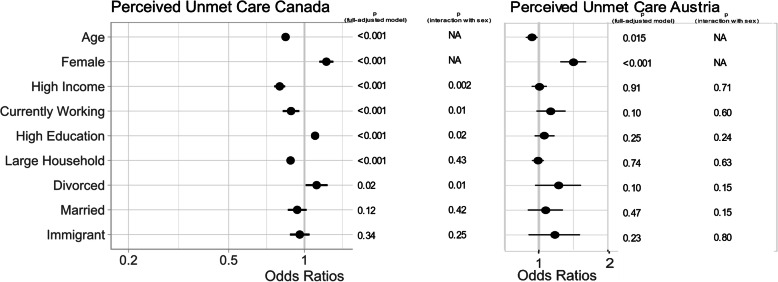
Forest plot of multivariate model for perceived unmet care in Canada using sex and all social variables, adjusted for age and significance of sex by social variable interaction terms

In both countries, perceived unmet care was positively associated with female sex (*p* < 0.04) (Fig. 2). In Canada, perceived unmet healthcare needs was negatively associated with working (*p* = 0.001), high income (*p* < 0.001) and large household size (p < 0.001) and positively associated with high education level. We also detected as significant interaction of sex with marital status (*p* = 0.04), income level (0.002), education level (*p* = 0.02) and working status (*p* = 0.01) in Canada. In Canada, being married decreased the likelihood of unmet healthcare needs and the effect was greater for males. Additionally, high income and working decreased the likelihood of unmet care needs, but the effect was greater for males, whereas the magnitude of the positive impact of high education on unmet care was stronger for females. In Austria, no significant impacts of any social determinants or their interactions with sex were detected.

## Discussion

This study leveraged two large national public health surveys representative of the countries’ respective populations to investigate the gendered impacts of social factors on self-reported health and access to care and to determine if these influences vary by country. We found that Canadians and Austrians overall reported high perceived health and low levels of unmet healthcare needs, however biological sex, many social determinants and their interactions with sex contributed to explaining these outcomes, implying gendered impacts of social determinants of health and access to care.

In both countries, we observed significant interaction terms between sex and social determinants on perceived health, demonstrating that these psychosocial variables impact male and females differently and reflect gendered inequalities. These findings highlight the importance of an intersectional approach to public health [11, 12], as many of these social determinants of health are studied in isolation, rather than considering how they may interact with each other. In both countries, marital status had gendered impacts on perceived health, potentially implying that gender roles and norms may impact an individual’s overall health. Marital status is often used as an estimate of social support which may influence overall health [15], however this effect is less pronounced in women, who may often have wider social support networks beyond their families [16] and is consistent with the results observed in both of our cohorts in which being divorced or widowed had a stronger negative effect on perceived health for males than females. Discrepancies in the relationship between marital status and mental health outcomes for men and women have also been observed, for example being divorced or widowed is associated with a higher rate of depression in men, whereas being married increased the risk for women [17].

We also found impacts of sex and of several socioeconomic indicators (working status, income and education level) on perceived health in both countries, and an interactive effect of education level and sex on perceived health in Canada. These findings are especially interesting considering that both countries are considered to have relatively high gender equality as measured by the UN Development Project’s Gender Inequality Index (GII) [18], which specifically uses educational attainment and labour force participation (though not income per se) as variables in its calculation of gender equality. We show that despite similar labour force participation and educational attainment between men and women, the health of males and females and the impacts of these variables on health between males and females vary, suggesting that gendered social norms persist and negatively impact health equity i.e. fair opportunity to achieve one’s full health potential. These findings highlight the importance of considering gender as an intersectional social variable when studying public health, however the differences we observed in results between countries indicate that country/culture should also be considered. Furthermore, the fact that high income, high education level and currently working were all positively independently associated with perceived heath in both countries, implies an influence of socioeconomic position on heath despite universal health insurance, which can be further confirmed by our results for perceived unmet care.

Both Canada and Austria have robust universal healthcare delivery systems [19, 20], designed to meet their populations’ basic healthcare needs regardless of socioeconomic position. Accordingly, we found the overall level of perceived unmet care was low in both countries. However, important inequalities in the social variables associated with perceived unmet care highlight that gap in access persist for vulnerable populations, and those cultural norms and policies may influence which populations are most vulnerable. In both countries, females were more likely to report unmet care and in Canada, we additionally observed gendered impacts of marital status and socioeconomic indicators (income level, education level and working status) on unmet care. This finding could also suggest a higher gender equality between sexes in Austria, which would be consistent with the country’s lower GII, but also indicates that sex and/or gender may be factors influencing perceived access to care in both countries, despite basic insurance coverage for all. Although the GII does include health-related variables in its calculation, both are related to maternity (specifically maternal mortality rate and rate of teen births) [18], and may therefore not account for all the healthcare needs of women and discrepancies with those of men. Further inequalities in perceived unmet care between countries indicate that country-specific differences in healthcare delivery systems may be important social determinants of health.

We found significant independent impacts of working status, household size and education status on perceived unmet care in Canada, whereas in Austria, we only observed impacts of sex and not of any social determinants. This finding could be due to differences in the healthcare delivery systems between countries. In Canada, a two-tiered health insurance system exists in which basic medically necessary needs are covered for all through provincial or territorial insurance, though the definition of medically necessary needs varies by province/territory. Additional needs (in many cases eye, dental and mental health care as well as prescription drugs) are covered by supplemental private insurance, often offered through employer benefits packages or paid out-of-pocket [20]. Our findings are consistent with other studies in Canada, including some on CCHS that conclude that low socioeconomic status is associated with poor health status and perceived access to care [21, 22], and additionally demonstrates that these impacts are gendered. In contrast, in Austria almost the entire population (99.9%) had health insurance coverage for all health care needs in 2011. The membership of a health insurance scheme is determined by occupation therefore, there is no competition between funds and the Austrian population enjoys above-average access to major medical-technical equipment, particularly in the area of computer tomography and magnetic resonance imaging [19]. On average people in Austria consulted a general practitioner, specialist physician or other social security contracted service provider 14 times in 2011 [19]. In a 2011 study, complaints of difficulty accessing services were only made by around 2% of the Austrian population, with just a very small proportion referring to barriers resulting from costs [19]. These differences in healthcare delivery systems could explain why we saw such large impacts of employment and income on perceived unmet care in Canada compared to Austria, and highlight the importance of considering social policies and cultural values when considering social determinants of health.

### Limitations

We recognize a few key limitations of this project. First, these surveys did not record gender identity, and as such we had to use sex as a proxy for gender identity even though those two variables are distinct and differ. We were therefore only able to compare results between males and females and were unable to determine the impacts of various gender identities on these outcomes or their intersection with other social determinants. Other studies have shown that people who do not identify as cis-men or cis-women often experience greater discrimination, higher psychosocial stress, poorer health outcomes and poor access to care than cis-gender men and women [23–26]. We suggest that surveys incorporate this distinction in the future to get a true picture of how gender identity may intersect with other social determinants to influence health. Similarly, we were unable to explore the intersection of race/ethnicity with sex or its independent impact on health because this variable was not collected in AT-HIS. However, race is known to be an important social variable that may independently impact health [27] and intersect with gender [28] and other social determinants. Additionally, CCHS excludes people living on reserves or crown land, thereby excluding primarily indigenous people and potentially those with the poorest access to care. We suggest that this information be collected in the future as it is very relevant to social determinants of health. A final limitation is that due to data sharing restrictions, we were unable to merge our databases and test for three-way interactions between sex, social determinants, and country, therefore our comparison of results between Austria and Canada is purely descriptive. Nevertheless, our exploration indicates that a formal investigation of the role of country/culture on perceived health would be an important future direction, ideally including many countries with more disparate GIIs and healthcare delivery systems.

## Conclusions

Patient-reported outcomes have been emerging as relevant indicators of individual well-being in clinical studies. Here, we show a significant interaction between sex and several social determinants (such as marital status, income and education level and working status) on perceived health and perceived unmet care, indicating gendered impacts of social determinants on people’s health experiences and emphasizing the importance of an intersectional approach to public health studies. Additionally, we show that results are country-specific, highlighting the important role that the social environment, in terms of cultural values, policies and lifestyle may play a role on overall health. We outline the need for further information to be collected in public health surveys (particularly gender as distinct from sex, and race/ethnicity and indigenous status) to gain a broader picture of these social determinants of health. Future work could also incorporate data from additional countries, particularly those with lower gender equality or with more distinctly different healthcare delivery systems.

## Supplementary Information

**Additional file 1.** GOING FWD Consortium Members not Listed as Authors.

## Data Availability

No original datasets were generated from this work. CCHS is owned by Statistics Canada and AT-HIS is owned by Statistik Austria, both of which are closed access. CCHS was accessed through the McGill-Concordia Laboratory of the Quebec Inter-University Centre for Social Statistics, and permission to use AT-HIS was granted directly by Statistik Austria.

## Abbreviations

CCHS: Canadian Community Health Survey
AT-HIS: Austrian Health Information Survey
PUMF: Public Use Microdata Files
