# Supplementary material for: Determinants of perceived health and unmet healthcare needs in universal healthcare systems with high gender equality
Source: BMC Public Health. 2021 Jul 31;21:1488. doi: 10.1186/s12889-021-11531-z (PMC8325202; doi:10.1186/s12889-021-11531-z)
Supplement: Supplementary file 1 — Additional file 1. GOING FWD Consortium Members not Listed as Authors. [file 12889_2021_11531_MOESM1_ESM.doc]

GOING FWD Consortium Members not Listed as Authors

| **Co-Investigators** | |
| --- | --- |
| Karin Humphries | University of British Columbia, Canada |
| Monica Parry | Lawrence S. Bloomberg Faculty of Nursing, Canada |
| Ruth Sapir-Pichhadze | McGill University Health Center and McGill University, Canada |
| Michal Abrahamowicz | McGill University Health Center and McGill University, Canada |
| Khaled El Emam | [University of Ottawa](https://research.uottawa.ca/people/el-emam-khaled), Canada |
| Simon Bacon | Concordia University, Canada |
| Peter Klimek | Medical University of Vienna, Austria |
| **Scientific Advisory Committee** | |
| Vera Regitz-Zagrosek | Charité – Universitätsmedizin Berlin, Germany |
| Londa Schiebinger | Stanford University, USA |
| Carole Claire | University of Lausanne, Switzerland |
| Rachel Dryer | Yale University, USA |
| **Trainees** | |
| Zahra Azizi | McGill University Health Center and McGill University, Canada |
| Uri Bender | McGill University Health Center and McGill University, Canada |
| Sabeena Jalal | McGill University Health Center and McGill University, Canada |
| Yumika Shiba | McGill University Health Center and McGill University, Canada |
| Alexia DellaVecchia | McGill University Health Center and McGill University, Canada |
| Pouria Alipour | McGill University Health Center and McGill University, Canada |
| Amanpreet Kaur | McGill University Health Center and McGill University, Canada |
| Divine-Favour Ofili | McGill University Health Center and McGill University, Canada |
| Zoe O'Neill | McGill University Health Center and McGill University, Canada |
| Rubee Dev | University of Alberta, Canada |
| Salima Hemani | Lawrence S. Bloomberg Faculty of Nursing, Canada |
| Heather Burnside | Lawrence S. Bloomberg Faculty of Nursing, Canada |
| Carola Deschinger | Medical University of Vienna, Austria |
| Juergen Harreiter | Medical University of Vienna, Austria |
| Simon Linder | Medical University of Vienna, Austria |
| Ann-Kristin Porth | Medical University of Vienna, Austria |
| Giulio Francesco Romiti | Sapienza University of Rome, Italy |
| Ange Laucyte-Cibulskiene | Karolinska Institutet, Sweden |
| Liam Ward | Karolinska Institutet, Sweden |
| Leah Muñoz | Karolinska Institutet, Sweden |
| Raquel Gomez De Leon | Universidad de Murcia, Spain |
| Ana Maria Lucas | Universidad de Murcia, Spain |
| Maria Sanchez | Universidad de Murcia, Spain |
| Raúl Nieto | Universidad de Murcia, Spain |
| Sandra Amador | Universidad de Murcia, Spain |
| Sonia Gayoso | Universidad de Murcia, Spain |
| **Patient Partners** | |
| Donna Hart | Ontario, Canada |
| Nicole Hartman/Nickerson | Nova Scotia, Canada |
| Angie Fullerton/MacCaul | PEI, Canada |
| Jeanette Smith | Ontario, Canada |
| Myra Lefkowitz | Ontario, Canada |
| Ann Keir | BC, Canada |
| Kyle Warkentin (caregiver) | BC, Canada |
| Rachael Manion | Ontario, Canada |
